# Time-efficient filtering of imaging polarimetric data by checking physical realizability of experimental Mueller matrices

**DOI:** 10.1093/bioinformatics/btae348

**Published:** 2024-06-03

**Authors:** Tatiana Novikova, Alexey Ovchinnikov, Gleb Pogudin, Jessica C Ramella-Roman

**Affiliations:** LPICM, CNRS, Ecole Polytechnique, IP Paris, Palaiseau, 91120, France; Department of Biomedical Engineering, Florida International University, Miami, FL , 33174, United States; Department of Mathematics, CUNY Queens College, 65-30 Kissena Blvd, Queens, NY, 11367, United States; Ph.D. Programs in Mathematics and Computer Science, CUNY Graduate Center, 365 Fifth Avenue, New York, NY, 10016, United States; Laboratoire d’informatique, CNRS, Ecole Polytechnique, IP Paris, Palaiseau, 91120, France; Department of Biomedical Engineering, Florida International University, Miami, FL , 33174, United States; Herbert Wertheim College of Medicine, Florida International University, Miami, FL, 33199, United States

## Abstract

**Motivation:**

Imaging Mueller polarimetry has already proved its potential for biomedicine, remote sensing, and metrology. The real-time applications of this modality require both video rate image acquisition and fast data post-processing algorithms. First, one must check the physical realizability of the experimental Mueller matrices in order to filter out non-physical data, i.e. to test the positive semi-definiteness of the 4 × 4 Hermitian coherency matrix calculated from the elements of corresponding Mueller matrix pixel-wise. For this purpose, we compared the execution time for the calculations of (i) eigenvalues, (ii) Cholesky decomposition, (iii) Sylvester’s criterion, and (iv) coefficients of the characteristic polynomial (two different approaches) of the Hermitian coherency matrix, all calculated for the experimental Mueller matrix images (600 pixels × 700 pixels) of mouse uterine cervix. The calculations were performed using C++ and Julia programming languages.

**Results:**

Our results showed the superiority of the algorithm (iv) based on the simplification via Pauli matrices over other algorithms for our dataset. The sequential implementation of latter algorithm on a single core already satisfies the requirements of real-time polarimetric imaging. This can be further amplified by the proposed parallelization (e.g. we achieve a 5-fold speed up on six cores).

**Availability and implementation:**

The source codes of the algorithms and experimental data are available at https://github.com/pogudingleb/mueller_matrices.

## 1 Introduction

Mueller polarimetry (MP) is an optical technique that is used for the characterization of polarimetric properties of a studied media for various applications such as medical imaging (Pierangelo *et al.* 2011, Vitkin *et al.* 2015, Lee *et al.* 2021, Bonaventura *et al.* 2022, Ramella-Roman and Novikova 2022), tissue engineering (Fricke *et al.* 2019, Ivanov *et al.* 2023), digital histology (Trifonyuk *et al.* 2020, Xia *et al.* 2020, Ushenko *et al.* 2021, Lee *et al.* 2022), remote sensing (Kattawar and Raković 1999, Novikova *et al.* 2010, Li *et al.* 2023), metrology (Kaplan *et al.* 2004, Fallet *et al.* 2011, Liu *et al.* 2015, Novikova and Bulkin 2021), material characterization (Hall *et al.* 2013, Arteaga and Kahr 2019), food quality control (Ignatenko *et al.* 2022), and many others. By measuring the changes in polarization states of incident light upon interaction with a sample, a MP system provides a set of 16 parameters that form a 4 × 4 real-valued transfer matrix [so-called Mueller matrix (MM)] (Azzam 1978, Goldstein 2010, Gil-Perez and Ossikovski 2022) of this sample. Each element of MM corresponds to a specific transformation of the input polarization states. Choosing an appropriate MM decomposition algorithm (Cloude 1986, Lu and Chipman 1996, Ghosh *et al.* 2009, Sheng *et al.* 2019, Li *et al.* 2020, Gil *et al.* 2023) allows researchers to extract and analyze quantitatively the depolarization, retardance, and diattenuation properties (Goldstein 2010) of a sample which carry relevant diagnostic or metrological information.

The design and calibration of MM polarimeters are usually optimized in terms of minimizing the measurement errors (Compain *et al.* 1999, Tyo 2002, De Martino *et al.* 2004, Bruce and Montes-González 2023). However, the presence of the residual systematic and random experimental errors may lead to nonphysical measurement results at certain measurement wavelengths or/and at certain image pixels, i.e. produce a particular 4 × 4 real-valued matrix that does not obey the condition of physical realizability for MM (see Section 2.1). A necessary and sufficient condition for the physical realizability of MM requires the corresponding 4 × 4 Hermitian coherency matrix (HCM) (Goldstein 2010) to be a positive semi-definite matrix (Cloude 1986, 1989). Thus, checking this condition for all acquired MMs represents a must step of the polarimetric data post-processing for filtering out nonphysical data.

In particular, the imaging MM polarimeters operating in either real (Lee *et al.* 2021, Ivanov *et al.* 2023) or reciprocal (Fallet *et al.* 2011) space produce a significant amount of polarimetric data at each single measurement, because the total number of image pixels is quite large (the resolution of CCD cameras is typically several hundreds of thousands pixels or more). Consequently, for the real-time imaging applications with a tight time budget it is important to find the most time-efficient algorithm for checking pixel-wise whether a 4 × 4 HCM is a positive semi-definite one.

The paper is organized as follows: in the next section, we describe the basics of Stokes–Mueller formalism and the MM images of mouse uterine cervix taken with the custom-built MM polarimetric system and used in our studies. Four different approaches for testing the physical realizability of the measured MMs, namely, the calculations of (i) eigenvalues, (ii) Cholesky decomposition, (iii) Sylvester’s criterion, and (iv) coefficients of the characteristic polynomial (two different approaches) of the corresponding HCM and the comparison of these algorithms in terms of the execution time and accuracy are presented and discussed in the third section of the paper. The last section summarizes our results and the perspectives of potential applications.

## 2 Materials and methods

### 2.1 Stokes–Mueller formalism

In this section, we briefly recall the theoretical framework for the description of fully or partially polarized light. Apart from light intensity and color the polarization of light reflects its vectorial nature. Let us consider the propagation of a plane polarized monochromatic electromagnetic (EM) wave through an infinite isotropic medium. By selecting a Cartesian coordinate system with *Oz* axis parallel to the direction of wave propagation ($\vec{k}=k\hat{\vec{z}}$, where k is a wave number), the oscillation of the electric field (EF) vector $\vec{E}$ at the point (0,0,z) is confined to the plane Ox-Oy orthogonal to Oz axis and is described by a superposition of two independent harmonic oscillators (Goldstein 2010)

(1) $$\begin{array}{c} E_{x}(z,t)=E_{x}^{0}\;\text{cos}(\omega t-kz+\delta_{x})\\ E_{y}(z,t)=E_{y}^{0}\;\text{cos}(\omega t-kz+\delta_{y}) \end{array},$$

where $E_{x}^{0}$ and $E_{y}^{0}$ are the constant amplitudes, *ω* is the angular frequency, k is the wavenumber, $\delta_{x}$ and $\delta_{y}$ are the arbitrary constant phases, and the subscripts x and y refer to the field components in the x- and y-directions, respectively. Equation (1) can be re-written as

(2) $${\left(\frac{E_{x}}{E_{x}^{0}}\right)}^{2}+{\left(\frac{E_{y}}{E_{y}^{0}}\right)}^{2}-2\frac{E_{x}E_{y}}{E_{x}^{0}E_{y}^{0}}\text{cos}\;\delta ={\;\text{sin}\;}^{2}\delta ,$$

where $\delta =\delta_{y}-\delta_{x}$ is the phase shift between the orthogonal transverse components of EF vector of a plane EM wave. Equation (2) describes a *polarization ellipse* that represents the trajectory of a tip of oscillating EF vector of polarized light. However, the direct observation of the polarization ellipse is impossible, because optical EF vector traces out the locus of points described by Equation (2) in a time interval of order $10^{-15}$ s.

For partially or completely depolarized light, the motion of EF vector is disordered in Ox-Oy plane and can be described by its probability distribution only. We cannot observe the amplitude of optical EF, but we can observe and measure the intensity of light. For linear optical systems, the measured intensity is obtained by averaging the square of EF amplitude and represents the second moments (quadratic quantities) of the EF distribution.

We denote the intensities related to the components of the EF vector parallel to Ox and Oy axes as $I_{x}=\langle E_{x}^{2}\rangle ,\;I_{y}=\langle E_{y}^{2}\rangle$, respectively. The angle brackets 〈·〉 indicate temporal, spatial, and spectral averaging, which depends on both sample and measurement conditions. We define four observable Stokes parameters as

(3) $$\begin{array}{c} S_{0}=0.5\langle E_{x}^{2}+E_{y}^{2}\rangle \\ S_{1}=0.5\langle E_{x}^{2}-E_{y}^{2}\rangle \\ S_{2}=\langle E_{x}^{2}E_{y}^{2}\;\text{cos}\;\delta \rangle \\ S_{3}=\langle E_{x}^{2}E_{y}^{2}\;\text{sin}\;\delta \rangle \end{array}.$$

The parameter $S_{0}$ is the total intensity of light, $S_{1}$ represents the difference in light intensities measured after a linear polarizer with optical axis aligned parallel with either Ox or Oy axes. The parameter $S_{2}$ is equivalent to the parameter $S_{1}$, but the intensities are measured after a linear polarizer with optical axis aligned at either +45° or −45°, respectively, within the plane orthogonal to the direction of light beam propagation. The parameter $S_{3}$ represents the difference between the intensities transmitted by either left or right circular polarizer. We define the Stokes vector as $\vec{S}={\left(S_{0},S_{1},S_{2},S_{3}\right)}^{T}$. The degree of polarization ρ of any Stokes vector $\vec{S}$ is defined as

(4) $$\rho =\frac{\sqrt{S_{1}^{2}+S_{2}^{2}+S_{3}^{2}}}{S_{0}},\;(0\leq \rho \leq 1),$$

where parameter ρ varies between 0, for totally depolarized light, and 1, for totally polarized light. Upon interaction with a sample the Stokes vector of incident light ${\vec{S}}^{\text{in}}$ undergoes a linear transformation, described by a 4×4 real-valued matrix M, which is called a Mueller matrix (MM) (Goldstein 2010) of a sample.

It is worth to mention that the measurements of an output Stokes vector may also provide information on a sample polarimetric properties (Ushenko *et al.* 2019, Peyvasteh *et al.* 2020, Iannucci *et al.* 2023), but contrary to MP data, an output Stokes vector will depend on the state of polarization of incident light beam.

(5) $$\left[\begin{array}{c} S_{0}^{\text{out}}\\ S_{1}^{\text{out}}\\ S_{2}^{\text{out}}\\ S_{3}^{\text{out}} \end{array}\right]=\left[\begin{array}{cccc} m_{00} & m_{01} & m_{02} & m_{03}\\ m_{10} & m_{11} & m_{12} & m_{13}\\ m_{20} & m_{21} & m_{22} & m_{23}\\ m_{30} & m_{31} & m_{32} & m_{33} \end{array}\right]\left[\begin{array}{c} S_{0}^{\text{in}}\\ S_{1}^{\text{in}}\\ S_{2}^{\text{in}}\\ S_{3}^{\text{in}} \end{array}\right].$$

It is obvious that physically realizable MM should transform any Stokes vector of incident light beam into a Stokes vector of output light beam with the degree of polarization less than or equal to 1. An Hermitian 4×4 coherency matrix H associated with matrix M is defined as (Gil-Perez and Ossikovski 2022):

(6) $$H=\frac{1}{4}\sum_{i,j=0}^{3}m_{ij}(\sigma_{i}⊗\sigma_{j}^{T}),$$

where ⊗ is the Kronecker product of matrices and $\sigma_{0},\ldots ,\sigma_{3}$ is an extended set of Pauli matrices (Feynman *et al.* 2006):

$$\sigma_{0}=I_{2},\sigma_{1}=\left(\begin{array}{cc} 1 & 0\\ 0 & -1 \end{array}\right),\sigma_{2}=\left(\begin{array}{cc} 0 & 1\\ 1 & 0 \end{array}\right),\sigma_{3}=\left(\begin{array}{cc} 0 & -i\\ i & 0 \end{array}\right).$$

It was demonstrated by Cloude (1986, 1989) that a 4 × 4 real-valued matrix M represents a physically realizable MM if and only if the associated HCM H calculated in Equation (6) is a positive semi-definite matrix. Due to the presence of measurement systematic errors and noise, this condition may not always be satisfied for the experimental polarimetric data. Thus, these data must undergo a physical realizability filtering before further data post-processing.

### 2.2 MM images of mouse uterine cervix

Imaging MP has proven its potential for various biomedical applications by helping the clinicians make better decisions about patient diagnosis and treatment (Pierangelo *et al.* 2011, Vizet *et al.* 2017, Qi and Elson 2017). The translation of this modality into clinical practice requires video rate image acquisition and the development and implementation of fast polarimetric data post-processing algorithms. As was mentioned above, the pixel-wise test of physical realizability of MM images is a first step of polarimetric data post-processing.

**Figure 1.**
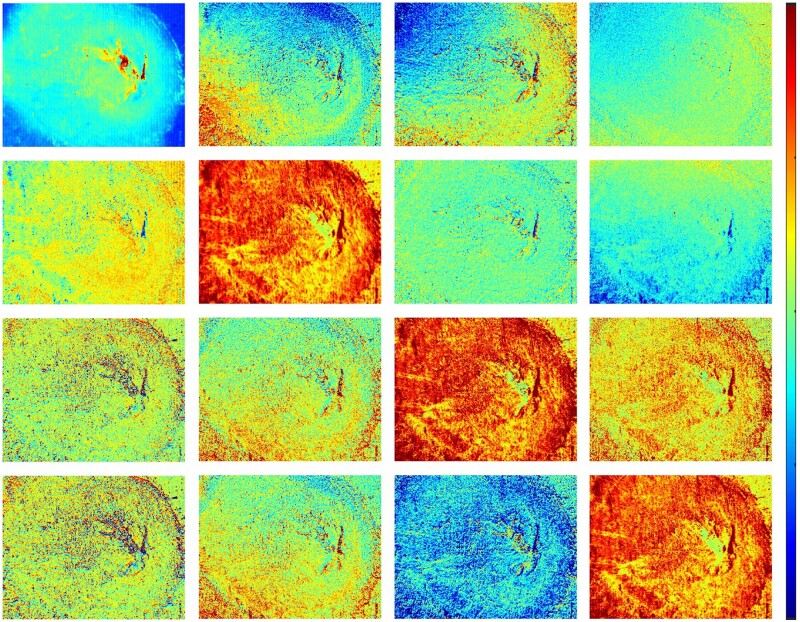
Mueller matrix images of mouse uterine cervix taken with a Mueller polarimeter. All elements of Mueller matrix (except of $m_{00}$) are normalized by *m*_00_ values pixel-wise. The color bar values vary within (i) [0;0.5] for $m_{00}$, (ii) [0;0.1] for all off-diagonal elements, and (iii) [0;0.2] for the elements $m_{11}$, $m_{22}$, $m_{33}$. The image size is 2 mm × 2.5 mm

In our studies, we used the experimental MM images (600 pixels × 700 pixels) of thin section (nominal thickness 50 μm) of mouse uterine cervix (Fig. 1). The research protocols were reviewed and approved by the Florida International University (registration number: IACUC-20–014).

These images were acquired with the custom-built imaging Mueller polarimetric system described in (Novikova and Ramella-Roman 2022). The modulation of incident light polarization and analysis of the detected signal was performed with the rotating quarter-wave plates and fixed polarizers inserted into both illumination and detection arm of the instrument. A 550-nm LED (Thorlabs, Newton, NJ) was used as a light source, a sCMOS camera (pco.edge by PCO, Kelheim, Germany) was used as a detector for the image registration. The system calibration was done with the eigenvalue calibration method described by Compain *et al.* (1999).

The experimental errors on the elements of measured MM of air (it should be a unity matrix) were below 1%. The complete description of the experimental setup can be found in (Novikova and Ramella-Roman 2022).

During data post-processing step, the check on physical realizability of each matrix from the dataset containing 420 000 (=600×700) matrices has to be performed first. Given the massive amount of polarimetric data to be tested, one needs to find the most time-efficient algorithm for checking the physical realizability of large dataset of MMs (i.e. positive semi-definiteness of the corresponding HCMs).

## 3 Tests on positive semi-definiteness of HCM

### 3.1 Algorithms

Definition 1. Hermitian n×n matrix H is positive semi-definite (or definite) if $(H\vec{x},\vec{x})\geq 0$ (or > 0 ) for all $\vec{x}\in C^{n}\backslash \{\vec{0}\}$.

We have tested four different approaches for checking the positive semi-definiteness (or positive definiteness) of HCM by calculating:

- eigenvalues of HCM,
- Cholesky decomposition of HCM,
- Sylvester’s criterion for HCM,
- coefficients of HCM characteristic polynomial.

It is worth considering both the pros and cons of each algorithm for filtering out nonphysical polarimetric data.

#### 3.1.1 HCM eigenvalues

Nowadays, the most common test for the physical realizability of a MM relies on the calculation of eigenvalues of the corresponding HCM (Cloude 1986, 1989, Gil 2016). All eigenvalues are nonnegative for a positive semi-definite matrix. Moreover, if MM of a sample is partially or completely depolarizing, the corresponding HCM must be a positive definite matrix. The latter comment is particularly relevant for almost all types of biological tissue because of strong light scattering within biotissue leading to the significant depolarization of detected scattered light (Tuchin 2007).

This method is widely spread, because one may not only filter out nonphysical data but also use a vector measure of depolarization based on the three smallest nonnegative eigenvalues as of the physically realizable MM. It was shown that such a metric may increase the polarimetric image contrast for certain classes of samples (Li *et al.* 2022, Sheppard *et al.* 2022, Gil *et al.* 2023). However, for a wide class of samples, it is not necessary to know the exact eigenvalues of HCM, when one needs to evaluate the sign of eigenvalues only.

#### 3.1.2 Cholesky decomposition of HCM

A decomposition of a Hermitian positive definite matrix H into the product of a lower triangular matrix L and its conjugate transpose matrix $L^{*}$ is called Cholesky decomposition: $H=LL^{*}$, and Cholesky decomposition is unique for such a matrix (Haddad 2009). To test whether a Hermitian matrix is positively definite using Cholesky decomposition, one needs to check whether the signs of all diagonal elements of the lower triangular matrix are positive. The fact that Cholesky decomposition may address the HCM positive definiteness only is not very restrictive for medical applications of MP, because, in general, biological tissues are highly depolarizing media and the corresponding HCMs are positive definite.

#### 3.1.3 Sylvester’s criterion

Sylvester’s criterion of positive definiteness of an arbitrary n×n Hermitian matrix states that this matrix is positive definite if and only if all its leading principal minors are positive (Cloude 1989, Gilbert 1991, Gil 2000). In case of a 4×4 HCM it means that all following matrices have a positive determinant:

- upper left 1×1 sub-matrix of HCM,
- upper left 2×2 sub-matrix of HCM,
- upper left 3×3 sub-matrix of HCM,
- HCM itself.

**Figure 2.**
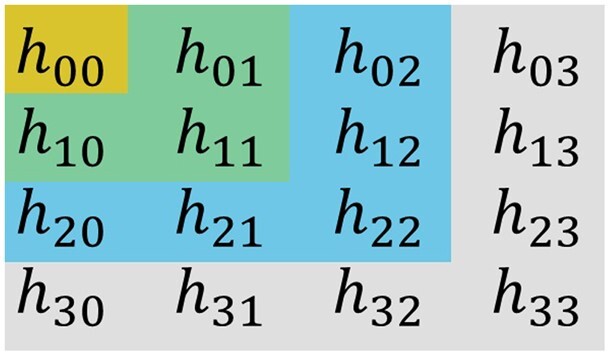
Illustration of the Sylvester’s criterion: calculations of leading principal minors of HCM, which are the determinants of the top left corner sub-matrices of 1×1, 2×2, 3×3, 4×4 dimensions.

The choice of corresponding elements of HCM for the calculations of leading principal minors is illustrated in Fig. 2. In the actual implementation, we applied (in advance) Maple’s command simplify to each of the determinant expressions to decrease the computing time.

#### 3.1.4 Coefficients of HCM characteristic polynomial

The characteristic polynomial (CP) of arbitrary n×n matrix H is defined as function $f(\lambda )=\det(H+\lambda I_{n})$, where $I_{n}$ represents the n×n identity matrix (Brooks 2006) (here we used “+” instead of the standard “–” in the definition of CP so that it will be easier to state the criterion). An arbitrary n×n matrix H with real eigenvalues is positive semi-definite if and only if all coefficients of its characteristic polynomial are nonnegative. This is true by applying Descartes’ rule of signs (Wang 2004) to f(λ) and using the equivalence between positive semi-definiteness and non-negativity of the eigenvalues.

It turns out that the actual presentation of the formulas for the coefficients of f(λ) can affect the computing time. In what follows, we use two versions for the presentation of the coefficients:

- formulas separately simplified in maple using command simplify
- formulas obtained using Pauli matrices (Feynman *et al.* 2006) as described below.

The coefficients of the CP of H are the elementary symmetric polynomials of its eigenvalues $\lambda_{1},\ldots ,\lambda_{4}$. We could find the values of these polynomials using the Newton identities (Macdonald 1995, p. 23) if we knew the values of the power sums $\lambda_{1}^{j}+\cdots +\lambda_{4}^{j}$ for j=1,…,4. These power sums, in turn, are exactly the traces $\text{tr}H,\ldots ,\text{tr}(H^{4})$. For computing these traces, we will use the following observations:

- Any power of H is again a linear combination of the form (6);
-
$\text{tr}(\sigma_{i}⊗\sigma_{j}^{T})=4$ if and only if i=j=0, and it is equal to 0 otherwise.

The latter property immediately gives us a simple formula $\text{tr}H=m_{00}$. For the second degree, we observe that the only way to obtain $I_{2}$ as a product of two Pauli matrices is to take a square. This implies that

$$\text{tr}(H^{2})=\frac{1}{16}\sum_{i,j=0}^{3}m_{ij}^{2}.$$

Using similar but much more tedious combinatorial considerations with Pauli matrices, we have obtained formulas for $\text{tr}(H^{3})$ and $\text{tr}(H^{4})$ as follows. Let $\tilde{M}$ denote the lower-right 3×3-submatrix of M. We start with $H^{3}$. We have

$$\text{tr}(H^{3})=\frac{3}{4}m_{00}\;\;\text{tr}(H^{2})-\frac{m_{00}^{3}}{8}-\frac{3}{8}\left(\text{det}\tilde{M}+\sum_{i=1}^{3}m_{i,0}\sum_{j=1}^{3}m_{0j}m_{ij}\right).$$

For $\text{tr}(H^{4})$, we will introduce some intermediate variables.

Let $S_{i}:=\sum_{j=1}^{3}m_{ij}^{2}$ for *i* = 1, 2, 3, and define

$$\begin{array}{c} A=\sum_{i=1}^{3}S_{i}^{2},\\ B=m_{10}^{2}(S_{2}+S_{3})+m_{20}^{2}(S_{1}+S_{3})+m_{30}^{2}(S_{1}+S_{2}). \end{array}$$

Then, for i<j<4, we set

$$P_{ij}=m_{i0}m_{j0}-{\left(-1\right)}^{\delta_{i0}}\sum_{k=1}^{3}m_{ik}m_{jk}$$

and define

$$C=-P_{01}^{2}-P_{02}^{2}-P_{03}^{3}+P_{12}^{2}+P_{13}^{2}+P_{23}^{2}.$$

We also define

$$D=\sum_{i=1}^{3}m_{0i}^{2},\;F=\sum_{i=1}^{3}m_{i0}^{2}.$$

Then, the final formula will be:

$$\begin{array}{c} \text{tr}(H^{4})=\frac{-\det(M)}{8}+\frac{3}{4}{\left(\text{tr}(H^{2})\right)}^{2}-\frac{A}{32}-\frac{B}{16}\\ -\frac{D}{16}\left(\frac{F}{2}+S_{1}+S_{2}+S_{3}\right)\\ +\frac{m_{00}}{4}\left(2\text{det}(\tilde{M})+\sum_{i=1}^{3}m_{i0}\sum_{j=1}^{3}m_{0j}m_{ij}\right)\\ -\frac{C}{16}-\frac{m_{00}^{2}F}{16}-\frac{1}{32}\left(m_{00}^{4}+m_{10}^{4}+m_{20}^{4}+m_{30}^{4}\right). \end{array}$$

The automatic verification of the formulas performed using Maple can be found in the repository (https://github.com/pogudingleb/mueller_matrices/blob/main/formula.mpl).

### 3.2 Comparison of the results: execution time and accuracy

We will start the comparison by outlining some common traits. The very problem is to check a particular property for each matrix in the dataset, so it consists of a large number of subproblems of fixed size, and we can see that:

- memory consumption in all the cases amounts simply to storing the input matrices (∼50 Mb), and the small additional amount of memory needed for processing a matrix does not depend on the total number of input matrices;
- time complexity of all the considered approaches is linear with respect to the number of matrices in the dataset;
- the algorithm is perfectly parallelizable, that is, the workload can be split almost evenly between several processing units. This is confirmed by the nearly linear speed-up we observe in the multi-core environment (see Table 1), which also makes it natural to expect good performance of this approach on GPUs.

**Table 1.** Execution time (milliseconds) for C++ implementation of the algorithms from Section 3.1 checking physical realizability of 420 000 Mueller matrices.

Algorithm\Used CPU cores	1	2	3	4	5	6
Eigen’s self-adjoint eigensolver	561.3	289.1	194.3	145.5	119.2	99.5
Eigen’s Cholesky decomposition	49.6	25.5	17.1	13.0	10.6	8.8
Sylvester’s criterion	34.6	17.7	11.9	9.0	7.4	6.3
Coefficients of CP (via maplesimplify)	26.8	13.5	9.1	6.9	5.6	4.7
Coefficients of CP (simplification via Pauli matrices)	13.1	6.6	4.5	3.5	2.9	2.6

To compare the *execution time and accuracy* of the algorithms from Section 3.1, we tested them on the experimental MM data described in Section 2.2. The results of the comparison in terms of the execution time depending on the number of used CPU cores are shown in Table 1. The code was written in C++, and the experiments were performed on a Mac computer with CPU 3.1 GHz 6-Core Intel Core i5 and 16 GB of RAM. For the eigenvalue computation and Cholesky decomposition, we used the numerical linear algebra library (http://eigen.tuxfamily.org, 2010, version 3.4.0). The C++ compiler was Clang 13.1.6, and the Mac OS version was 12.3. The runtime values were averaged over 20 runs; the standard deviation was 1.6% or lower. The implementation is available at: https://github.com/pogudingleb/mueller_matrices.

The number of filtered out nonphysical matrices is 868 and constitutes approximately 0.2% of the total number of matrices for all tested algorithms, thus, proving the good quality of our experimental polarimetric data. In addition, all nonphysical data were found at the same image pixels by all algorithms. So, all the algorithms were numerically stable enough for the considered data. Further applications in a broader range of datasets may require additional numerical error analysis.

Our computational experiments confirm that the method of calculations of HCM eigenvalues for checking positive definiteness (semi-definiteness) of HCM is orders of magnitude slower and more memory demanding compared to other three methods.

We achieve an additional speed-up by parallelization. Indeed, the matrix for each pixel can be processed independently of the other pixels. On our 6-core environment, we achieved a run-time speed-up of approximately 5.5 times over the sequential computation. This is more than enough for the processing of the matrices to be used in real-time.

For comparison, we also ran this in Julia version 1.10.0 in CentOS Linux 7, on Intel(R) Xeon(R) CPU E5-2680 v2 @ 2.80 GHz, with 20 cores, and found out that the relative placement of the algorithms in terms of runtime is the same as that of our C++ code. Our Julia implementations of the algorithms via Sylvester’s criterion and via coefficients of the CP are also sufficiently fast to be used in real time. The detailed results are reported at: https://github.com/pogudingleb/mueller_matrices/blob/main/src_julia/julia_code.out.

## 4 Conclusion

Our results on filtering the imaging polarimetric data by checking pixel-wise physical realizability of the experimental Mueller matrices of mouse uterine cervix demonstrated the superiority (in terms of execution time) of the algorithm of calculations of the coefficients of the characteristic polynomial of HCM via Pauli matrices for our dataset compared to other considered algorithms, whereas keeping the same accuracy. It is quite logical that the filtering method based on the calculations of HCM eigenvalues may not be optimal in terms of execution time, because we generate redundant data, i.e. the absolute values of the eigenvalues of HCM, that are not used for MM filtering.

Another explanation for the observed performance gap is related to the fact that general-purpose linear algebra software is often not optimized for the matrices of small dimensions (e.g. 4×4), which we consider in this paper.

Our findings are important for many applications of MP that generate significant amount of polarimetric data and require both real-time data acquisition and fast data post-processing algorithms, in particular, for imaging MP used for (i) *in vivo* medical diagnosis and surgery guidance in clinical settings, (ii) metrology in semi-conductor industry, and (iii) remote sensing.

In this paper, we limited ourselves to the approaches that are based on matrix-by-matrix computations. While such methods are easy to parallelize and analyze, taking advantage of the similarity between the matrices at adjoining time points when acquiring time series data would allow to improve the efficiency even further.

## Supplementary Material

btae348_Supplementary_Data
