# Supplementary material for: Time-efficient filtering of imaging polarimetric data by checking physical realizability of experimental Mueller matrices
Source: Bioinformatics. 2024 Jun 3;40(7):btae348. doi: 10.1093/bioinformatics/btae348 (PMC11283285; doi:10.1093/bioinformatics/btae348)
Supplement: btae348_Supplementary_Data [file btae348_supplementary_data.pdf]

A. Appendix

In this appendix, we will provide explicit formulas for $\operatorname{tr}(\mathbf{H}^3)$ and $\operatorname{tr}(\mathbf{H}^4)$, which we obtained through combinatorial considerations with Pauli matrices. Let $\widetilde{\mathbf{M}}$ denote the lower-right 3×3-submatrix of $\mathbf{M}$.

We start with $\mathbf{H}^3$. We have

$$\operatorname{tr}(\mathbf{H}^3) = \frac{3}{4} m_{00} \operatorname{tr}(\mathbf{H}^2) - \frac{m_{00}^3}{8} - \frac{3}{8}\left(\det \widetilde{\mathbf{M}} + \sum_{i=1}^{3} m_{i,0} \sum_{j=1}^{3} m_{0j} m_{ij}\right).$$

For $\operatorname{tr}(\mathbf{H}^4)$, we will introduce some intermediate variables. Let $S_i := \sum_{j=1}^{3} m_{ij}^2$ for $i = 1, 2, 3$, and define

$$A = \sum_{i=1}^{3} S_i^2,$$
$$B = m_{10}^2(S_2 + S_3) + m_{20}^2(S_1 + S_3) + m_{30}^2(S_1 + S_2).$$

Then, for $i < j < 4$, we set

$$P_{ij} = m_{i0} m_{j0} - (-1)^{\delta_{i0}} \sum_{k=1}^{3} m_{ik} m_{jk}$$

and define

$$C = -P_{01}^2 - P_{02}^2 - P_{03}^3 + P_{12}^2 + P_{13}^2 + P_{23}^2.$$

We also define

$$D = \sum_{i=1}^{3} m_{0i}^2, \quad F = \sum_{i=1}^{3} m_{i0}^2.$$

Then the final formula will be:

$$\begin{aligned}\operatorname{tr}(\mathbf{H}^4) = \frac{-\det(\mathbf{M})}{8} + \frac{3}{4}\left(\operatorname{tr}(\mathbf{H}^2)\right)^2 - \frac{A}{32} - \frac{B}{16} \\ - \frac{D}{16}\left(\frac{F}{2} + S_1 + S_2 + S_3\right) \\ + \frac{m_{00}}{4}\left(2\det(\widetilde{\mathbf{M}}) + \sum_{i=1}^{3} m_{i,0} \sum_{j=1}^{3} m_{0j} m_{ij}\right) \\ - \frac{C}{16} - \frac{m_{00}^2 F}{16} - \frac{1}{32}\left(m_{00}^4 + m_{10}^4 + m_{20}^4 + m_{30}^4\right).\end{aligned}$$

The automatic verification of the formulas performed using MAPLE can be found in the repository `https://github.com/pogudingleb/mueller_matrices/blob/main/formula.mpl`.
